# Eco-certification and greening the Brazilian soy and corn supply chains

**DOI:** 10.1088/1748-9326/9/3/031002

**Published:** 2014-03-04

**Authors:** Leah K VanWey, Peter D Richards

**Affiliations:** 1 Environmental Change Initiative, Brown University, Providence, RI 02912, USA; 2 Department of Sociology, Brown University, Providence, RI 02912, USA; 3 Population Studies and Training Center, Brown University, Providence, RI 02912, USA

## Abstract

Garrett *et al*’s recent letter (2013 *Environ. Res. Lett.* **8** 044055) shows the trade value of Brazil’s production of non-genetically modified (GM) crops, and argues that production for this niche market laid the foundation for the expansion of a variety of non-GM and eco-certification systems. We argue that the conditions underlying the development and perpetuation of the non-GM certification systems are transient. The expansion of soy production has dampened the conditions that promoted the dominance of non-GM soy in the region. The state at the heart of the production of conventional soy, Mato Grosso, already has transitioned to almost 90% GM soy in the most recent agricultural season. The continued viability of eco-certification systems depends on strengthening institutions on the demand side, and ensuring farm-level costs on the supply side match price premiums reaching the farm level.

Garrett *et al*’s (2013) recent letter, ‘Globalization’s unexpected impact on soybean production in South America: linkages between preferences for non-genetically modified crops, eco-certifications, and land use’, shows a positive empirical relationship between a country’s production of non-genetically-modified crops and trade patterns with key European and Asian countries where consumers prefer such crops. It then argues that Brazil’s specialization in non-GM crops resulted in an upgraded supply chain that laid the foundation for eco-certification programs certifying crops as ecologically responsible but not non-GM. The evidence is compelling, and shows the potential for nation-scale consumer preferences to effect positive conservation behavior in a distant agricultural frontier, fulfilling the conservation promise of large-scale farming noted by Nepstad *et al* (2006). Yet we argue that changes in the structural conditions underlying Brazil’s agricultural sector, including increased access and new infrastructure channels, are increasingly opening areas in Brazil to the cultivation of GM soybeans. In turn, this has reduced the area and size of Brazil’s non-GM crop harvests and called into question not only the future of non-GM programs in Brazil, both also the future of environmental certification systems. The conditions that allowed Brazil and especially Mato Grosso to develop its niche and capture much of the global market for non-GM soybean production were transient. For these to have had lasting impacts, we argue that certification systems need stronger institutionalization, that transaction costs for certification must be decreased, especially for small and medium-sized farms, and a greater non-GM price premium must come to farmers. These lessons could be applied to eco-certification programs in the future.

Garrett *et al* highlight the concentration of non-GM production, primarily in the State of Mato Grosso, and ProTerra certification, primarily among a few large landholders. Mato Grosso, located in the southern Amazon region, and largely segmented from the core agricultural areas in southern and southeastern Brazil, was uniquely positioned for the production of non-GM crops. First, GM seeds, which were first introduced to southern Brazil from Argentina, were not initially suited for the region and offered lower yields in the low latitudes and acidic soils of Mato Grosso. Second, the geographic isolation of the state also encouraged different export routes, which facilitated the development of an infrastructure for shipping and storing exclusively non-GM soybeans, much of which were exported directly to Europe or Asia. However, at the farm level, the hegemony of non-GM varieties was a result, in part, of asymmetric markets and the abilities of a limited number of seed purchasers in the supply chain to control farmers’ production decisions. In this sense, companies such as Cargill or Brazil’s Maggi Group could leverage their market advantage to ensure that local farmers would plant non-GM soybeans by either refusing purchase of GM varieties or paying lower prices. This market structure is similar to the structure of Brazil’s noted soybean moratorium in newly deforested farmland, a private policy initiative that relies on the coercion of downstream purchasers to enforce controls on environmental practices. While this system effectively passes on production costs to farm-level producers, it does not necessarily pass on a profit premium needed to encourage farmers to grow these varieties. Instead, the price premium for GM free soybeans was absorbed into the supply chain and processing facilities, rather than at the farm level. Comparisons of price data for GM soybeans in Campo Novo de Parecis with prices for non-GM varieties in nearby Sapezal, both in western Mato Grosso, show little or no farm-level price premium for non-GM (IMEA 2012). One result of this market structure, and one which Garrett *et al* highlight, is that today the majority of non-GM soy production is concentrated in the few large landholders who also control their own supply chains.

The geographic segregation of production areas that once facilitated the segregated production and supply chains for GM and non-GM crops, and the maintenance of asymmetric markets that enabled soybean purchasers to control farmers’ planting decisions have eroded with the development and proliferation of Mato Grosso’s soybean sector. Increasingly, farmers in traditionally non-GM zones can now find buyers for GM crops, a trend that is evident in the declining acreage of certified non-GM soybeans in Brazil. Brazil’s volume of Cert-ID certified soybeans fell from a high of more than seven million tons in 2005 to approximately five million tons between 2008 and 2013 (Cert ID 2013). The volume of ProTerra (see Garrett *et al*) soybeans has also fallen from 4.5 to 4 million tons from 2007 to 2013. Estimates suggest that today more than 90% of Brazil’s soybeans are transgenic (Celeres 2013). Even in Mato Grosso, once the home to large quantities of conventional, non-GM soybean farms, the production of non-GM soybeans as a percent of total harvest has fallen every year since 2006. Even here, GM representation is rapidly approaching ninety percent (IMEA 2013). One result is that Cert-ID and ProTerra soy are quickly losing importance in a discussion of the future of the region.

For farmers to invest in eco-certification, a larger price premium will need to come to the farm level, to compensate for the added cost of consumer preferred farming practices, rather than be captured by crushers and distributors. The higher prices will be necessary to motivate farmers to pursue the cost of certification, and to have the extra capital available to bring their farms into compliance through reforestation, documentation, and other labor consuming management practices. It remains to be seen whether middlemen or even farmers will see a value in purchasing eco-certified soy and corn at a premium in Brazil. However, such a move could lay the groundwork for farmers to increase the supply of eco-certified crops.

On the demand side, a price premium depends on a market for the certified product. The move from non-GM certification to eco-certification fundamentally changes the nature of that market. Garrett *et al* suggest that the institutional development on the supply side that took place to monitor the GM status of supply chains is potentially enough to ensure a continued green supply chain for soy. It is beyond the scope of their analysis to study the demand side of the equation, the creation of a market of buyers willing to pay a premium for eco-certified soy or corn. Literature on certification systems shows the importance of such market creation, especially for a product that is perceived as functionally equivalent to the end user (Dubuisson-Quellier 2013, Manning *et al* 2012). The non-GM certification entailed a different type of consumer preference, one in which the end user perceives the product to be functionally superior. Consumers in Europe and their governments identified GM crops as dangerous to health and therefore created a clear market for certified non-GM crops through labeling laws that allowed individual consumers and governments to implement their preferences (European Commission 2000). In contrast, the eco-certified crops are more similar to certified sustainable timber (Bartley 2007) or to renewable energy (Vasi 2011); the end product is functionally equivalent but some consumers have preferences for different production conditions. At the moment there is little demand for eco-certified soy or corn; grains and oilseeds are traditionally differentiated on oil and protein yields, not the behaviors that underlie their production; and most of it ends up as feed in industrial-scale animal raising facilities, where the preferences of individual consumers are a small part of the purchase decision. Either social movement pressure or government labeling laws for meats (allowing the expression of individual consumer preferences) will need to create demand among animal raising facilities for eco-certified soy and corn.

Garrett *et al* suggest that the upgrading of supply chains in the country is both testament to the power of consumer preference and the foundation for continued eco-certification programs (independent of the move away from non-GM soy). We discuss here the passing of the non-GM moment in Brazil, and pose two challenges to eco-certification programs. On the demand side, markets and institutions must be created anew for eco-certified soy and corn in the same way that government labeling laws and consumer preferences in Europe created the market for non-GM certified soy. On the supply side, the widespread production of eco-certified crops depends on the incentives and costs of certification for farmers and their ability to meet the conditions of eco-certification. These both depend in turn on how much of the price premium for certified crops is passed to them. The non-GM certification case does not bode well for processors and exporters passing a price premium to farmers in the future. However, their willingness depends in turn on the level of demand, suggesting that future research on greening soy, corn, and meat supply chains will have much to tell us about the potential future of eco-certification in Brazil.
